# Factors associated with vaccination coverage: a population-based survey in Vitória, Espírito Santo, Brazil, 2020-2021

**DOI:** 10.1590/S2237-96222024v33e20231195.especial2.en

**Published:** 2025-01-10

**Authors:** João Paulo Cola, Laylla Ribeiro Macedo, Mariana Pereira da Silva Araújo, Thiago Nascimento do Prado, Tatiane Comerio, Adriana Ilha da Silva, Ana Paula França, José Cássio de Moraes, Ethel Leonor Noia Maciel, ICV 2020 Group, Adriana Ilha da Silva, Alberto Novaes Ramos, Ana Paula França, Andrea de Nazaré Marvão Oliveira, Antonio Fernando Boing, Carla Magda Allan Santos Domingues, Consuelo Silva de Oliveira, Ethel Leonor Noia Maciel, Ione Aquemi Guibu, Isabelle Ribeiro Barbosa Mirabal, Jaqueline Caracas Barbosa, Jaqueline Costa Lima, José Cássio de Moraes, Karin Regina Luhm, Karlla Antonieta Amorim Caetano, Luisa Helena de Oliveira Lima, Maria Bernadete de Cerqueira Antunes, Maria da Gloria Teixeira, Maria Denise de Castro Teixeira, Maria Fernanda de Sousa Oliveira Borges, Rejane Christine de Sousa Queiroz, Ricardo Queiroz Gurgel, Rita Barradas Barata, Roberta Nogueira Calandrini de Azevedo, Sandra Maria do Valle Leone de Oliveira, Sheila Araújo Teles, Silvana Granado Nogueira da Gama, Sotero Serrate Mengue, Taynãna César Simões, Valdir Nascimento, Wildo Navegantes de Araújo

**Affiliations:** 1 Universidade Federal do Espírito Santo, Laboratório de Epidemiologia, Vitória, ES, Brazil; 2 Santa Casa de São Paulo, Faculdade de Ciências Médicas, São Paulo, SP, Brazil; Universidade Federal do Espírito Santo, Vitória, ES, Brazil; Universidade Federal do Ceará, Departamento de Saúde Comunitária, Fortaleza, CE, Brazil; Faculdade Ciências Médicas Santa Casa de São Paulo, São Paulo, SP, Brazil; Secretaria de Estado da Saúde do Amapá, Macapá, AP, Brazil; Universidade Federal de Santa Catarina, SC, Brazil; Organização Pan-Americana da Saúde, Brasília, DF, Brazil; Instituto Evandro Chagas, Belém, PA, Brazil; Faculdade de Ciências Médicas Santa Casa de São Paulo, Departamento de Saúde Coletiva, São Paulo, SP, Brazil; Universidade Federal de Mato Grosso, Cuiabá, MT, Brazil; Universidade Federal do Paraná, Curitiba, PR, Brazil; Universidade Federal de Goiás, Goiânia, GO, Brazil; Universidade Federal do Piauí, Teresina, PI, Brazil; Universidade de Pernambuco, Faculdade de Ciências Médicas, Recife, PE, Brazil; Instituto de Saúde Coletiva, Universidade Federal da Bahia, Salvador, BA, Brazil; Secretaria de Estado da Saúde de Alagoas, Maceió, AL, Brazil; Universidade Federal do Acre, Rio Branco, AC, Brazil; Universidade Federal do Maranhão, Departamento de Saúde Pública, São Luís, MA, Brazil; Universidade Federal de Sergipe, Aracaju, SE, Brazil; Secretaria Municipal de Saúde, Boa Vista, RR, Brazil; Fundação Oswaldo Cruz, Mato Grosso do Sul, Campo Grande, MS, Brazil; Fundação Oswaldo Cruz, Escola Nacional de Saúde Pública Sergio Arouca, Rio de Janeiro, RJ, Brazil; Universidade Federal do Rio Grande do Sul, Porto Alegre, RS, Brazil; Fundação Oswaldo Cruz, Instituto de Pesquisa René Rachou, Belo Horizonte, MG, Brazil; Secretaria de Desenvolvimento Ambiental de Rondônia, Porto Velho, RO, Brazil; Universidade de Brasília, Brasília, DF, Brazil

**Keywords:** Cobertura de Vacunación, Vacunas, Inmunización, Salud Infantil, Encuestas de Población, Vaccination Coverage, Vaccines, Immunization, Child Health, Population Surveys

## Abstract

**Objective:**

To estimate prevalence of the full vaccination schedule for children 12 to 24 months old and to analyze associated factors.

**Methods:**

Survey with cluster sampling carried out in Vitória, Espírito Santo, Brazil, between December 16, 2020, and January 4, 2021. Children born in Vitória in 2017 and 2018 were included. We estimated the prevalence of vaccination schedules. Poisson regression was used to verify association with full vaccination coverage.

**Results:**

We included 788 children. Full vaccination coverage was found to be 57% taking a 95% confidence interval (95%CI 50.98;62.98). Prevalence of full vaccination coverage was lowest when private services were used for immunization (prevalence ratio [PR] 0.67; 95%CI 0.51;0.86) and when mothers had ≥ 4 children (PR 0.55; 95%CI 0.32;0.94).

**Conclusion:**

We found low vaccination coverage and a drop in booster doses. Use of private services for immunization and number of children were associated with incomplete vaccination coverage.

## Study contributions

**Main results:**

Full vaccination coverage was found for just over half of the population, with a decrease for vaccines that require a booster dose. Use of private services for immunization and greater number of children were negatively associated with full vaccination coverage.

**Implications for services:**

The need to strengthen active tracing of children whose vaccinations are overdue within health service territories, especially for families with a larger number of children and those using private services.

**Perspectives:**

The need for innovative health service strategies, to increase childhood vaccination coverage, especially to address insecurity and fear of vaccination, resulting from disinformation.

## RESUMEN

**Objetivo:**

Estimar la prevalencia del esquema vacunal completo de 12-24 meses de edad y analizar los factores asociados.

**Métodos:**

Encuesta con muestreo por conglomerados realizada en Vitória, Espírito Santo, entre el 16 de diciembre/2020 y el 4 de enero/2021. Se incluyeron 788 niños nacidos en Vitória en 2017-2018, se estimó la prevalencia de los esquemas vacunales. Se utilizó la regresión de Poisson para verificar la asociación con cobertura vacunal.

**Resultados:**

La cobertura vacunal fue 57%, con un intervalo de confianza del 95% (IC_95_% 50,98;62,98). El uso de un servicio privado vacunal (razón de prevalencia [RP] 0,67; IC_95_% 0,51;0,86) y madre con ≥ 4 hijos (RP 0,55; IC_95_% 0,32;0,94) tuvieron la prevalencia más baja de cobertura vacunal completa.

**Conclusión:**

Encontramos baja cobertura vacunal con reducción de dosis de refuerzo. El uso de servicio privado para vacunación y el número de hijos se asoció con cobertura de vacunación incompleta.

## INTRODUCTION

Immunization is one of the main disease prevention measures.^1^ The consolidation of the National Immunization Program (*Programa Nacional de Imunizações* - PNI) in Brazil was achieved through the elimination of rubella and its congenital syndrome, neonatal tetanus, eradication of polio and reduced transmission of diphtheria, tetanus and pertussis.^1,2^ The PNI has made progress with the control, reduction and elimination of diseases, changing the epidemiological scenario of vaccine-preventable diseases in Brazil.^1,2^

The PNI currently provides more than 20 routine vaccines, covering all life cycles. In the case of children up to 2 years old, 15 immunizations are available to prevent more than 19 diseases.^3^ However, in recent years, Brazil has recorded considerable reductions in vaccination coverage in the child population, and has not achieved the targets recommended by the PNI.^4^ In view of all the efforts to increase vaccination coverage nationwide, when comparing coverage in 2022 and 2023, hepatitis A vaccination coverage increased from 73% to 79.5%. The pneumococcal conjugate vaccine (PCV10) booster rose from 71.5% to 78.0% in 2023. The inactivated polio vaccine 1, 2 and 3 (IPV) reached 74.6% coverage, compared to 67.1 % in 2022. However, it still remains below the target recommended by the PNI.^5^

In the municipality of Vitória, coverage of the measles, mumps and rubella (MMR) vaccine fell from 92.33%, in 2022, to 88.27% in 2023; meningococcal serogroup C vaccination (MENC) for children under 1 year of age fell from 87.25% in 2022 to 85.24% in 2023; while the IPV vaccine remained at 91% in both those years. Furthermore, coverage of the first PCV10 booster fell from 80.94% in 2022 to 78.59% in 2023.^6^

Dissemination of fake information about immunization, misinformation and lack of knowledge about the severity of vaccine-preventable diseases can trigger low adherence to vaccination, thus reducing vaccination coverage.^1,2,4,7,8^ Factors related to maternal, child and family context characteristics can influence vaccination coverage, as can factors related to health service structure and lack of supplies.^4,7-10^ The relevance of the topic and the scientific need for epidemiological studies, with more robust methodological approaches, justify this study, the objective of which was to estimate prevalence of the full vaccination schedule in children 12 to 24 months old and to analyze associated factors, in Vitória, Espírito Santo, Brazil.

## METHODS

### Study design and background 

This was a population-based survey with cluster sampling carried out in the municipality of Vitória, capital of the state of Espírito Santo, Brazil, between December 16, 2020 and January 4, 2021. The study is part of the 2020 National Vaccination Coverage Survey.^11^

Vitória has an estimated population of 365,855 inhabitants, of which 5.88% (21,406) are children between 0 and 4 years old. In December 2020, the municipality had 78 teams working in the Family Health Strategy (*Estratégia de Saúde da Família* - ESF), with 74.32% ESF coverage and 93.8% Primary Care coverage. 

At that time there were 43 vaccination rooms in the city: 28 located in public health centers; one in a Reference Center for Special Immunobiological Products; 12 private vaccination services and two maternity hospitals that administer the first dose of BCG and hepatitis B (HepB) to newborn babies.

### Participants

The study population was made up of the 2017 and 2018 live birth cohorts, registered on the Live Birth Information System (*Sistema de Informações sobre Nascidos Vivos* - SINASC), totaling 9,252 live births. The inclusion criteria were children born in the city of Vitória during the years 2017 and 2018 who had a vaccination card. The exclusion criteria were children who changed municipalities, deaths and children with mothers under 19 years of age. 

### Sample size 

The parameters used to calculate the sample size were an estimated 70% vaccination coverage prevalence, 5% estimation error, 95% confidence interval (95%CI) and design effect due to the use of clusters equal to 1.4.

Sampling took place by socioeconomic strata, using the city’s urban census tracts, according to the 2010 Demographic Census. After identifying the socioeconomic strata, an estimate of children residing in each census tract was made, by georeferencing their addresses held on the SINASC. The projection was based on the distribution found by the 2010 Census. The census tracts were systematically selected at random so as to cover the entire geographic area. The interviewers traveled around the area to find the children who lived there, until the pre-established number for each stratum was reached.^11^

The sampling process and calculation details of the original survey are available in the methodological protocol of the 2020 National Vaccination Coverage Survey.^11^ For this study, the power of the sample recruited was calculated based on the prevalence of vaccination coverage found at 24 months of age, considering a 5% alpha error. The calculation was performed using Stata v. 14.0. 

### Data collection 

Data collection took place through interviews with the children’s parents or guardians, using a structured questionnaire, during which the children’s vaccination cards were photographed using an electronic device. The interviewers were trained and validated to administer the questionnaire and take the photographs. Vaccination card dates were subsequently input by two trained and validated professionals with experience in immunization, based on the photographs of the vaccination cards. 

The questionnaire consisted of the following blocks: sociodemographic data of the children, reproductive data of the mothers, household and family consumption, and the children’s vaccination data. With regard to immunization data, we used the 2015 Ministry of Health vaccination schedule, as follows:

- At birth: BCG and HepB
- 2 months: 1^st^ dose of diphtheria, tetanus, pertussis, hepatitis B, *Haemophilus influenzae* b (DTP-Hib-HepB), IPV 1^st^ dose, PVC10 1^st^ dose and oral rotavirus vaccine (RV1) 1^st^ dose;
- 3 months: MENC 1^st^ dose;
- 4 months: DTP-Hib-HepB2^nd^ dose, IPV2^nd^ dose, PCV10 2^nd^ dose and RV12^nd^ dose;
- 5 months: MENC2^nd^ dose;
- 6 months: DTP-Hib-HepB3^rd^ dose, IPV 3^rd^ dose;
- 12 months: 1^st^ PCV10 booster, MMR 1^st^ dose and hepatitis A (HepA) 1^st^ dose;
- 15 months: diphtheria, tetanus and pertussis (DTP) vaccine1^st^ dose, 1^st^ poliovirus 1 and 3 (attenuated) (bOPV) booster, 1^st^ MENC booster, MMR 2^nd^ dose and a single dose of varicella vaccine.

### Variables

The study’s dependent variable was the full vaccination schedule (yes; no). Children who received all the doses recommended by the Ministry of Health’s vaccination schedule, regardless of the date of administration, were considered to have full vaccination coverage.

The independent variables were grouped into:

Family characteristics

- Bolsa *Família* Program beneficiaries (yes; no);
- Use of a private immunization service (yes; no);
- Household crowding (yes, ≥ 4 people per bedroom; no, ≤ 3 people per bedroom);
- Lives with grandmother (yes; no);
- Family income (in BRL: up to BRL 1000; BRL 1001-3000; BRL 3001-8000; ≥ BRL 8001).

Maternal characteristics
Age (19-29 years; 30-39 years; ≥ 40 years);
Schooling (in years of study: ≤ 8 years; 9-12 years; 13-15 years; ≥ 16 years);
Race/skin color (White; Black; mixed race; Asian/Indigenous);
Paid job (yes; no);
Number of children (1 child; 2 children; 3 children; ≥ 4 children);
Lives with a partner (yes; no);
Type of delivery (vaginal; cesarean).

Child characteristics
Sex (male; female);
Race/skin color (White; Black; mixed race; Asian/Indigenous);
Attends daycare or nursery (yes; no);
Birth order (first-born; second-born; third-born; ≥ fourth-born).

### Statistical analysis 

Due to the sample being stratified and clustered by census tracts, sampling weights were calculated for each household interviewed, in order to enable unbiased estimation of the parameters of interest in the population, in addition to the use of disproportionate allocation procedures. The steps for calculating and calibrating the weights are detailed in the operational article about the National Survey.^11^ Statistical analyses were performed using Stata v. 14.0. As the sampling design was considered complex, we used Stata’s set of svyset commands, which reduces underestimation of point estimation variance.^12^

Prevalence of full vaccination schedule up to 24 months of life and for each immunizing agent was estimated using a 95% confidence interval (95%CI). The relative and absolute frequencies of the independent variables were also calculated using the full vaccination coverage variable. 

In order to verify association of independent variables with prevalence of full vaccination coverage, we calculated prevalence ratios (PR) and 95%CIs using robust variance Poisson regression. Initially, we performed bivariate (crude) analysis, followed by hierarchical multiple analysis, in which all variables at each level were input to the model at the same time and were kept in the following levels as adjustments, if the p-value ≤0.05. 

In the multiple analysis, the following groups of variables were taken as hierarchical levels, respectively: family characteristics, maternal characteristics and child characteristics.

### Ethical aspects 

The study was approved by the Research Ethics Committee of the *Instituto de Saúde Coletiva da Universidade Federal da Bahia*, as per Opinion No. 3.366.818, on June 4, 2019, and Certificate of Submission for Ethical Appraisal (*Certificado de Apresentação de Apreciação* Ética - CAAE) No. 4306919.5.0000.5030; and by the Research Ethics Committee of the *Irmandade da Santa Casa de São Paulo*, as per Opinion No. 4.380.019, on November 4, 2020, and CAAE No. 39412020.0.0000.5479

All participants were instructed regarding the research procedures and expressed their free and unimpeded desire to participate in the study, by signing the informed consent form.

## RESULTS

The vaccination survey sample calculation resulted in 904 children. After the sample selection and interviews process, 788 children participated in the survey – a loss of 12.8%. Vaccination coverage, considering all immunobiological products at 12 months old, was 66.2% (95%CI 57.83;73.67), while full vaccination coverage at 24 months old was 57% ( 95%CI 50.98;62.98). Taking the prevalence found at 24 months old, the sample power was 97%.

In Table 1, it can be seen that vaccination coverage was below 90% for all immunobiological products assessed, with the first doses of DTP-Hib-HepB (95%CI 78.21;94.66), PCV10 (95%CI 78.22;94.67) and IPV (95%CI 77.95;94.44) showing the greatest prevalence, with 88% of the children vaccinated with the first dose, while the first PCV10 booster (72.4%, 95%CI 61.76;81.03) and the first bOPV booster (78.3%, 95%CI 66.46;86.89), and the second MMR dose (71.3%, 95%CI 61.41;79.61), showed lower vaccination coverage prevalence. 

**Table 1:** Prevalence (%) and 95% confidence interval (95%CI) of the full vaccination schedule at 12 and 24 months old among children born alive in 2017 and 2018, Vitória, Espírito Santo, Brazil (n = 572)

Vaccines	Doses	(95%CI)
BCG	Single dose	79.77 (68.13;87.91)
Hepatitis B	Initial dose	78.97 (67.20;87.31)
Diphtheria, tetanus, pertussis, hepatitis B and *Haemophilus influenzae* B	1^st^ Dose	88.86 (78.21;94.66)
Inactivated poliovirus 1, 2 and 3	1^st^ Dose	88.57 (77.95;94.44)
Pneumococcal conjugate	1^st^ Dose	88.87 (78.22;94.67)
Rotavirus	1^st^ Dose	85.92 (75.26;92.44)
Meningococcal serogroup C	1^st^ Dose	87.96 (77.31;94.00)
Diphtheria, tetanus, pertussis, hepatitis B and *Haemophilus influenzae* B	2^nd^ Dose	85.49 (74.95;92.07)
Inactivated poliovirus 1, 2 and 3	2^nd^ Dose	85.56 (75.01;92.12)
Pneumococcal conjugate	2^nd^ Dose	76.94 (64.56;85.94)
Rotavirus	2^nd^ Dose	80.68 (69.13;88.92)
Meningococcal serogroup C	2^nd^ Dose	85.06 (74.36;91.79)
Diphtheria, tetanus, pertussis, hepatitis B and Haemophilus *influenzae* B	3^rd^ Dose	81.80 (70.39;89.48)
Inactivated poliovirus 1, 2 and 3	3^rd^ Dose	82.08 (70.68;89.69)
Pneumococcal conjugate	1^st^ Booster	72.43 (61.76;81.03)
Meningococcal serogroup C	1^st^ Booster	79.63 (67.92;87.83)
Measles, mumps and rubella	1^st^ Dose	87.06 (76.52;93.29)
Hepatitis A	Single dose	84.63 (73.37;91.67)
Measles, mumps and rubella	2^nd^ Dose	71.37 (61.41;79.61)
Poliovirus 1 and 3 (attenuated)	1^st^ Booster	78.37 (66.46;86.89)
Diphtheria, tetanus e pertussis	1^st^ Booster	77.88 (65.95;86.48)
Varicella	Initial Dose	84.89 (73.66;91.86)
Vaccination coverage at 12 months old	–	66.20 (57.83;73.67)
Vaccination coverage at 24 months old	–	57.09 (50.98;62.98)

Regarding the characteristics of the families, it can be seen that the majority (57.2%, 95%CI 41.99;71.17) used private vaccination services, did not live in homes with household crowding (95.1%, 95% CI 91.10;96.63), were not *Bolsa Família* Program beneficiaries (83.6%, 95%CI 75.69;89.31), the majority of the children did not live with their grandmother (83.7%, 95%CI 73.94;90.91) and monthly family income was equal to or greater than BRL 8001 (40.6%, 95%CI 17.54;58.77) (Table 2). When analyzing these characteristics, considering vaccination coverage status categorized as *full* and *incomplete*, a similar trend to that described for the total number of children studied can be seen, although notable differences were non-use of private vaccination services and monthly family income between BRL 1001 and BRL 3000 for the group with full vaccination coverage (Table 2).

**Table 2:** Distribution of prevalence (%) and 95% confidence interval (95%CI) of the full vaccination schedule at 12 and 24 months old, according to the family characteristics of children born alive in 2017 and 2018, Vitória, Espírito Santo, Brazil (n =572 )

Variable	Vaccination coverage					
	**Incomplete**		**Full**		**Total**	
	**n**	**% (95%CI)**	**n**	**% (95%CI)**	**n**	**% (95%CI)**
**Private service vaccination**						
No	121	13.12 (8.61;19.49)	230	29.63 (20.07;41.38)	351	42.75 (28.78;57.92)
Yes	175	29.68 (21.57;39.31)	258	27.57 (19.92;36.80)	433	57.25 (41.99;71.17)
**Household crowding**						
No	281	41.28 (35.00;47.65)	463	54.00 (48.40;59.51)	744	95.18 (91.10;96.63)
Yes	17	1.73 (0.91;3.26)	23	3.09 (1.69;5.58)	40	4.82 (3.37;8.90)
*Bolsa Família* **Program beneficiary**						
No	252	38.51 (32.00;45.47)	393	45.14 (39.55;50.85)	645	83.65 (75.69;89.31)
Yes	47	4.42 (2.75;7.03)	95	11.93 (7.55;18.35)	142	16.35 (10.66;24.25)
**Lives with grandmother**						
No	256	35.58 (28.76;43.50)	404	47.92 (43.00;52.88)	660	83.71 (73.94;90.91)
Yes	42	7.07 (3.07;15.45)	85	9.21 (5.72;14.52)	127	16.29 (9.72;25.97)
**Monthly family income (BRL)**						
≤ 1000	42	5.36 (3.08;9.17)	79	12.36 (7.01;20.87)	121	17.72 (9.48;24.21)
1001-3000	44	5.18 (2.95;8.97)	108	15.58 (9.88;23.72)	154	20.77 (12.21;26.06)
3001-8000	74	10.65 (6.35;17.32)	108	10.18 (6.19;16.30)	182	20.83 (12.13;26.36)
≥ 8001	98	17.43 (8.10;33.57)	161	23.25 (14.39;35.32)	259	40.68 (17.54;58.77)

With regard to maternal characteristics, there was greater prevalence of mothers aged between 30 and 39 years (56.7%, 95%CI 40.97;71.28), with 16 or more years of schooling (63%, 95%CI 48.47;74.41), of White race/skin color (50.8%, 95%CI 44.96;56.64), with a paid job (67.3%, 95%CI 54.22;78.20), only one child (47.1%, 95%CI 41.42;53.03), living with a partner (83.4%, 95%CI 75.52;89.23 ) and who had a cesarean section (71%, 95%CI 59.09;80.64). When comparing the full vaccination coverage and incomplete coverage columns, similar characteristics can be seen between the two groups (Table 3)

**Table 3:** Distribution of prevalence (%) and 95% confidence interval (95%CI) of the full vaccination schedule at 12 and 24 months old, according to the characteristics of mothers of children born alive in 2017 and 2018 in Vitória, Espírito Santo, Brazil (n = 572)

Variable	Vaccination coverage					
	**Incomplete**		**Full**		**Total**	
	**n**	**% (95%CI)**	**n**	**% (95%CI)**	**n**	**% (95%CI)**
**Age (years)**						
19-29	61	7.39 (4.68;11.48)	108	15.75 (10.00;23.92)	169	23.14 (15.30;33.41)
30-39	167	26.52 (18.35;36.67)	255	30.25 (22.76;38.96)	422	56.76 (40.97;71.28)
≥ 40	71	9.03 (4.52;17.24)	125	11.07 (7.23;16.59)	196	20.10 (12.60;30.52)
**Schooling (years)**						
≤ 8	12	1.08 (0.51;2.26)	20	1.82 (0.98;3.34)	32	2.89 (1.64;5.04)
9-12	33	4.04 (2.32;6.95)	47	6.71 (3.93;11.21)	80	10.75 (6.67;16.85)
13-15	57	5.60 (3.44;8.99)	127	17.76 (11.29;26.81)	184	23.36 (15.34;33.83)
≥ 16	195	32.49 (24.76;41.30)	287	30.51 (23.12;39.07)	482	63.00 (48.47;74.41)
**Race/skin color**						
White	168	21.49 (13.04;33.32)	239	29.32 (21.82;38.13)	407	50.81 (44.96;56.64)
Black	37	4.24 (2.44;7.28)	64	7.36 (4.51;11.80)	101	11.61 (7.45;17.65)
Mixed race	92	17.47 (8.78;31.77)	170	19.38 (12.89;28.08)	262	36.85 (30.66;43.50)
Asian/Indigenous	1	0.003 (0.001;0.23)	8	0.07 (0.02;1.68)	9	0.73 (0.31;1.71)
**Paid job**						
No	103	11.77 (7.64;17.71)	194	20.88 (13.89;30.17)	297	32.66 (21.79;45.76)
Yes	195	31.46 (23.78;40.31)	287	35.88 (28.67;43.79)	482	67.34 (54.22;78.20)
**Number of children**						
1	127	17.34 (10.18;27.96)	208	29.85 (22.21;38.80)	335	47.19 (41.42;53.03)
2	119	20.09 (11.67;32.36)	198	19.21 (12.87;27.66)	317	39.29 (33.09;45.86)
3	33	3.51 (2.01;6.08)	54	5.37 (3.31;8.58)	87	8.87 (5.66;13.64)
≥ 4	20	2.00 (0.94;4.21)	28	2.65 (1.44;4.84)	48	4.65 (2.75;7.75)
**Lives with a partner**						
No	50	5.68 (3.39;9.36)	86	10.84 (7.01;16.38)	136	16.51 (10.77;24.48)
Yes	247	37.52 (30.07;44.87)	397	45.96 (40.66;51.36)	644	83.49 (75.52;89.23)
**Type of delivery**						
Cesarean	194	32.51 (24.81;41.28)	323	38.53 (32.65;44.76)	517	71.04 (59.09;80.64)
Vaginal	101	10.24 (6.54;15.68)	166	18.72 (12.52;27.04)	267	28.96 (19.36;40.91)

Taking the total number of children studied, it can be seen that the majority were male (53.3%, 95%CI 48.01;58.54), of White race/skin color (55.8%, 95%CI 49.21;62.19), attended daycare (76%, 95%CI 64.72;84.58) and were first-born (55.1%, 95%CI 49.57;60 ,63). No differences were found in the children’s characteristics between the full vaccination coverage and incomplete coverage groups, or in relation to the total number of children assessed (Table 4).

**Table 4:** Distribution of prevalence (%) and 95% confidence interval (95%CI) of the full vaccination schedule at 12 and 24 months old, according to the characteristics of children born alive in 2017 and 2018, Vitória, Espírito Santo, Brazil (n = 572)

Variable	Vaccination coverage					
	**Incomplete**		**Full**		**Total**	
	**n**	**% (95%CI)**	**n**	**% (95%CI)**	**n**	**% (95%CI)**
**Sex**						
Female	137	16.71 (9.99;26.62)	228	29.98 (22.58;38.59)	365	46.69 (41.46;51.99)
Male	162	26.20 (17.82;36.77)	261	27.11 (18.41;37.99)	423	53.31 (48.01;58.54)
**Race/skin color**						
White	185	22.22 (13.68;34.01)	283	33.58 (26.79;41.12)	468	55.80 (49.21;6219)
Black	16	2.14 (1.04;4.32)	46	4.97 (2.93;8.31)	62	7.11 (4.38;11.33)
Mixed race	97	18.47 (9.79;32.11)	156	18.36 (12.04;26.99)	253	36.83 (29.98;44.26)
Asian/Indigenous	1	0.01 (0.01;0.73)	3	0.16 (0.01;0.55)	4	0.26 (0.04;0.78)
**Attends daycare**						
No	63	7.59 (4.39;12.81)	83	16.38 (7.77;31.30)	146	23.97 (15.42;35.28)
Yes	236	35.32 (28.14;43.23)	406	40.71 (27.40;55.53)	642	76.03 (64.72;84.58)
**Birth order**						
First-born	167	20.32 (12.38;31.53)	263	34.84 (28.01;42.36)	430	55.16 (49.57;60.63)
Second-born	89	17.65 (9.09;31.47)	162	16.18 (10.77;23.59)	251	33.82 (26.99;41.40)
Third-born	24	2.86 (1.53;5.28)	39	3.92 (2.36;6.43)	63	6.77 (4.25;10.64)
≥ Fourth-born	18	1.85 (0.86;3.93)	24	2.39 (1.26;4.51)	42	4.24 (2.48;7.16)

The crude analysis of family, mother and child characteristics showed significant association with full vaccination coverage for the following factors: use of private immunization services (PR 0.53; 95%CI 0.40;0.69), being a *Bolsa Família* Program beneficiary (PR 1.28; 95%CI 1.11;1.46), have monthly family income ≥ BRL 8001 (PR 0.66; 95%CI 0.53;0.82 ), maternal age 30-49 years (PR 0.68; 95%CI 0.55;0.84) and ≥ 40 years (PR 0.65; 95%CI 0.46;0.92), having a paid job (PR 0.76; 95%CI 0.62;0.94), and vaginal child delivery (PR 1.23; 95%CI 1.04;1.47). In the hierarchical multiple analysis, full vaccination coverage was associated with use of private immunization services (PR 0.66; 95%CI 0.51;0.86) and mothers having three children (PR 0.67; 95%CI 0.45;0.98) or ≥ four children (PR 0.55; 95%CI 0.32;0.94) (Table 5).

**Table 5:** Prevalence ratio (PR) and confidence interval (95%CI) of the full vaccination schedule at 12 and 24 months old, according to the variables used in the study with children born alive in 2017 and 2018 in Vitória, Espírito Santo, Brazil

Variable	PR Crude model (95%CI)	p-value	PR Multilevel model (95%CI)	p-value
Family characteristics				
**Private service vaccination**		< 0.001		0.002
No	1.00		1.00	
Yes	0.53 (0.40;0.69)		0.66 (0.51;0.86)	
**Household crowding**		0.392		0.644
No	1.00		1.00	
Yes	1.14 (0.84;1.54)		0.94 (0.73;1.20)	
*Bolsa Família* **Program beneficiary**		< 0.001		0.720
No	1.00		1.00	
Yes	1.28 (1.11;1.46)		1.02 (0.89;1.16)	
**Lives with grandmother**		0.663		0.496
No	1.00		1.00	
Yes	1.05 (0.82;1.33)		1.05 (0.90;1.23)	
**Monthly family income (BRL)**		< 0.001		0.065
≤ 1000	1.00		1.00	
1001-3000	1.08 (0.87;1.35)		1.12 (0.89;1.40)	
3001-8000	0.63 (0.45;1.35)		0.75 (0.54;1.04)	
≥ 8001	0.66 (0.53;0.82)		0.95 (0.68;1.32)	
**Maternal characteristics**				
**Age (years)**		< 0.001		0.153
19-29	1.00		1.00	
30-49	0.68 (0.55;0.84)		1.24 (0.95;1.62)	
≥ 40	0.65 (0.46;0.92)		1.25 (0.89;1.75)	
**Schooling (years)**		< 0.001		0.078
≤ 8	1.00		1.00	
9-12	1.03 (0.73;1.44)		0.98 (0.67;1.44)	
13-15	1.21 (0.90;1.62)		1.07 (0.74;1.55)	
≥ 16	0.62 (0.43;0.88)		0.65 (0.40;1.05)	
**Race/skin color**		0.082		0.637
White	1.00		1.00	
Black	1.22 (0.79;1.87)		0.82 (0.57;1.18)	
Mixed race	0.93 (0.41;2.12)		0.79 (0.46;1.36)	
Asian/Indigenous	1.48 (0.86;2.56)		0.97 (0.52;1.83)	
**Paid job**		0.012		0.303
No	1.00		1.00	
Yes	0.76 (0.62;0.94)		0.90 (0.73;1.09)	
**Number of children**		0.491		0.045
1	1.00		1.00	
2	0.72 (0.35;1.49)		0.70 (0.39;1.25)	
3	1.02 (0.69;1.51)		0.67 (0.45;0.98)	
≥ 4	0.96 (0.58;1.58)		0.55 (0.32;0.94)	
**Lives with a partner**		0.003		0.096
No	1.00		1.00	
Yes	0.76 (0.64;0.91)		1.13 (0.97;1.30)	
**Type of delivery**		0.014		0.928
Cesarean	1.00		1.00	
Vaginal	1.23 (1.04;1.47)		1.01 (0.82;1.30)	
**Child characteristics**				
**Sex**		0.390		0.280
Female	1.00		1.00	
Male	0.76 (0.41;1.41)		0.83 (0.59;1.16)	
**Race/skin color**		0.257		0.584
White	1.00		1.00	
Black	1.26 (0.84;1.87)		0.82 (0.58;1.15)	
Mixed race	0.88 (0.41;1.88)		0.81 (0.58;1.14)	
Asian/Indigenous	1.17 (0.46;2.99)		1.13 (0.41;3.13)	
**Attends daycare**		0.340		0.339
No	1.00		1.00	
Yes	0.73 (0.11;2.52)		0.69 (0.42;1.13)	
**Birth order**		0.786		0.218
First-born	1.00		1.00	
Second-born	0.75 (0.35;1.62)		0.82 (0.55;1.22)	
Third-born	1.00 (0.67;1.49)		0.68 (0.45;1.03)	
≥ Fourth-born	0.99 (0.60;1.61)		0.71 (0.46;1.10)	

## DISCUSSION

Our study showed low vaccination coverage among children born alive in 2017 and 2018 in the municipality of Vitória. Vaccination coverage showed a relevant drop among those over one year old. We also found that the first doses of the DTP-Hib-HepB, PCV10 and IPV vaccines had the highest coverage in that population; on the other hand, the first PCV10 and bOPV boosters and the second MMR dose had the lowest vaccination coverage. Regarding factors associated with full vaccination coverage, use of private immunization services and having a greater number of children were negatively associated with full vaccination coverage.

The study’s limitations include the COVID-19 pandemic, which made access to the homes of families selected by the survey difficult, as well as their fear of infection by letting the study team into their homes. To this end we used social media and also gave interviews about the survey to the main local media outlets. The difficulty in accessing households may have affected a higher percentage of a given social stratum of the population. However, we believe that, after adjusting the model, this was not found to interfere in the result. Furthermore, the study design, the child sampling process and the sample size enabled us to detect differences between the groups studied. 

The results of the survey described vaccination coverage well below the 95% coverage target established by the World Health Organization and agreed to by the Brazilian PNI.^3,4,13^ In this regard, low vaccination coverage can lead to the reemergence and worsening of diseases that had been controlled and eliminated, such as measles, the reemergence of which Brazil has been facing since 2018.^14-16^

An ecological study, carried out with secondary data, described an inversely proportional correlation between the increase in the number of measles cases and the decrease in vaccination coverage in Brazil.^17^ In 2018, in Brazil, measles vaccination coverage was 67%, and there were 10,326 cases of the disease.^17^ Therefore, the low measles vaccine coverage, found in our study, demonstrates an emerging scenario of occurrence of autochthonous cases of the disease. 

 The results of this study show a considerable drop in the third IPV dose in relation to the first dose, which suggests that coverage tends to be lower in vaccines that require a booster, compared to single dose vaccines.^18^ In the national vaccination survey, all municipalities studied showed a drop in coverage of vaccines that require more than one dose, when comparing the first dose with the others.^11^

The results of our study showed a lower chance of full vaccination coverage in children who use private vaccination services. A possible explanation can be considered to be the occurrence of strategic activities and the initiatives that are introduced to keep vaccination rooms open during the entire public health center opening hours; avoiding access barriers, such as not requiring proof of residence for vaccination, whereby just having a Brazilian National Health System card is sufficient; taking advantage of vaccination opportunities, such as consultations or other procedures at health centers, to check vaccination status, in addition to vaccination campaigns, which contribute to the implementation of vaccination coverage widely recommended by the PNI in primary health care centers; and, undoubtedly, the contribution of the ESF in actively tracing children whose vaccination is overdue.^19-21^

We also highlight the continuity of the universal supply of vaccines in the public network and their facilitated access for the population, given the extensive national network of primary health care services, together with other surveillance actions, which are fundamental and essential for achieving the objective of eliminating and controlling diseases such as smallpox, diphtheria, polio and measles.^4^

Children from families with higher income had the lowest prevalence of full vaccination records in the crude analysis, as opposed to families that received some form of government assistance. Children from families with lower purchasing power live in more peripheral neighborhoods with good ESF coverage in Vitória. Therefore, we believe that the ESF and government aid are important health strategies and public policies for promoting vaccination.^22,23^ In a global scenario of fake news about vaccines, the ESF plays a fundamental role in combating misinformation and actively tracing children whose vaccinations are overdue, especially through home visits homes by community health agents.^24,25^

Regarding receipt of government aid, one must also consider the evident risk of an increase in the reduction of vaccination coverage, due to changes in the period immediately prior to 2023 in the conditions associated with social aid, which separated receipt of the benefit, renamed *Auxílio Brasil* at the time, from several conditions required to be met by parents and guardians, including up-to-date vaccination, according to the PNI, of children who make up the immediate family, leading to discouragement of vaccination and risk of reduced vaccination coverage.

In our study, having three or more children was negatively associated with full vaccination coverage. We believe that this lower prevalence of full vaccination may be due to the mother/guardian having less time available for full care of the child and, clearly, when a family gets bigger and has more expenses, taking care of health and prioritizing it can decline.^26^

Although the study investigated above all the relationship between mothers and child vaccination coverage, it did not ignore a relevant gender perspective regarding this choice of method. Responsibility for child care is the duty of all those in charge of children, as set forth in the Federal Constitution and other specific legislation, whereby the mother, father or any other person who acts as a guardian are responsible for providing health care for the child in question, such as adherence to vaccination campaigns.^27,28^ However, in the same way that it is important to recognize that, in legislative terms, there is an equal obligation between fathers and mothers to take responsibility for the care – and, therefore, for vaccination – of children, it is also important to recognize that we still live in a structurally sexist society, which causes an overlapping responsibility of roles for women, especially when they are mothers, culturally placing the social and community duty of fulfilling parental care on them.^29,30^

This study enabled low vaccination coverage to be identified, as well as the decrease in coverage of vaccines that require a booster dose, and indicates, epidemiologically, factors associated with full vaccination coverage. Therefore, the findings allow us to stress the need for innovative strategies to increase childhood vaccination coverage, especially to address insecurity and fear of vaccination, resulting from disinformation. In this sense, community health agents need to be trained to actively trace children whose vaccinations are overdue within health service territories, especially in the case of families with a larger number of children, those who use private services and those with lower income, as well as disseminating positive information about vaccination, in addition to helping to combat false information about vaccination. 
