# Supplementary material for: Factors associated with vaccination coverage: a population-based survey in Vitória, Espírito Santo, Brazil, 2020-2021
Source: Epidemiol Serv Saude. 2025 Jan 10;33(spe2):e20231195. doi: 10.1590/S2237-96222024v33e20231195.especial2.en (PMC11734598; doi:10.1590/S2237-96222024v33e20231195.especial2.en)
Supplement: Translation

# Translation: Fatores associados ao esquema completo de vacinação: inquérito de base populacional em Vitória, Espírito Santo, 2020-2021

**DOI:** 10.1590/S2237-96222024v33e20231195.especial2.pt

**Authors:** João Paulo Cola, Laylla Ribeiro Macedo, Mariana Pereira da Silva Araújo, Thiago Nascimento do Prado, Tatiane Comerio, Adriana Ilha da Silva, Ana Paula França, José Cássio de Moraes, Ethel Leonor Noia Maciel, Grupo ICV 2020, Adriana Ilha da Silva, Alberto Novaes Ramos, Ana Paula França, Andrea de Nazaré Marvão Oliveira, Antonio Fernando Boing, Carla Magda Allan Santos Domingues, Consuelo Silva de Oliveira, Ethel Leonor Noia Maciel, Ione Aquemi Guibu, Isabelle Ribeiro Barbosa Mirabal, Jaqueline Caracas Barbosa, Jaqueline Costa Lima, José Cássio de Moraes, Karin Regina Luhm, Karlla Antonieta Amorim Caetano, Luisa Helena de Oliveira Lima, Maria Bernadete de Cerqueira Antunes, Maria da Gloria Teixeira, Maria Denise de Castro Teixeira, Maria Fernanda de Sousa Oliveira Borges, Rejane Christine de Sousa Queiroz, Ricardo Queiroz Gurgel, Rita Barradas Barata, Roberta Nogueira Calandrini de Azevedo, Sandra Maria do Valle Leone de Oliveira, Sheila Araújo Teles, Silvana Granado Nogueira da Gama, Sotero Serrate Mengue, Taynãna César Simões, Valdir Nascimento, Wildo Navegantes de Araújo

**Affiliations:** 1 Universidade Federal do Espírito Santo, Laboratório de Epidemiologia, Vitória, ES, Brasil; 2 Santa Casa de São Paulo, Faculdade de Ciências Médicas, São Paulo, SP, Brasil; Universidade Federal do Espírito Santo, Vitória, ES, Brasil; Universidade Federal do Ceará, Departamento de Saúde Comunitária, Fortaleza, CE, Brasil; Faculdade Ciências Médicas Santa Casa de São Paulo, São Paulo, SP, Brasil; Secretaria de Estado da Saúde do Amapá, Macapá, AP, Brasil; Universidade Federal de Santa Catarina, SC, Brasil; Organização Pan-Americana da Saúde, Brasília, DF, Brasil; Instituto Evandro Chagas, Belém, PA, Brasil; Faculdade de Ciências Médicas Santa Casa de São Paulo, Departamento de Saúde Coletiva, São Paulo, SP, Brasil; Universidade Federal de Mato Grosso, Cuiabá, MT, Brasil; Universidade Federal do Paraná, Curitiba, PR, Brasil; Universidade Federal de Goiás, Goiânia, GO, Brasil; Universidade Federal do Piauí, Teresina, PI, Brasil; Universidade de Pernambuco, Faculdade de Ciências Médicas, Recife, PE, Brasil; Instituto de Saúde Coletiva, Universidade Federal da Bahia, Salvador, BA, Brasil; Secretaria de Estado da Saúde de Alagoas, Maceió, AL, Brasil; Universidade Federal do Acre, Rio Branco, AC, Brasil; Universidade Federal do Maranhão, Departamento de Saúde Pública, São Luís, MA, Brasil; Universidade Federal de Sergipe, Aracaju, SE, Brasil; Secretaria Municipal de Saúde, Boa Vista, RR, Brasil; Fundação Oswaldo Cruz, Mato Grosso do Sul, Campo Grande, MS, Brasil; Fundação Oswaldo Cruz, Escola Nacional de Saúde Pública Sergio Arouca, Rio de Janeiro, RJ, Brasil; Universidade Federal do Rio Grande do Sul, Porto Alegre, RS, Brasil; Fundação Oswaldo Cruz, Instituto de Pesquisa René Rachou, Belo Horizonte, MG, Brasil; Secretaria de Desenvolvimento Ambiental de Rondônia, Porto Velho, RO, Brasil; Universidade de Brasília, Brasília, DF, Brasil

**Keywords:** Cobertura Vacinal, Vacinas, Imunização, Saúde da Criança, Inquéritos Populacionais

## Abstract

**Objetivo:**

Estimar a prevalência do esquema vacinal completo dos 12 aos 24 meses de vida e analisar os fatores associados ao esquema vacinal completo.

**Métodos:**

Inquérito com amostragem por conglomerados realizado em Vitória, Espírito Santo, entre 16 de dezembro de 2020 e 4 de janeiro de 2021. Foram incluídas crianças nascidas em Vitória nos anos de 2017 e 2018. Estimamos as prevalências dos esquemas vacinais. Utilizou-se regressão de Poisson para verificar a associação com a cobertura vacinal.

**Resultados:**

Incluímos 788 crianças. A cobertura vacinal foi de 57% [intervalo de confiança de 95% (IC_95_%) 50,98;62,98]. Utilizar serviço privado para imunização [razão de prevalência (RP) 0,67; IC_95_% 0,51;0,86] e mãe ter ≥ 4 filhos (RP 0,55; IC_95_% 0,32;0,94) apresentaram as menores prevalências de cobertura vacinal completa.

**Conclusão:**

Encontramos baixa cobertura vacinal com diminuição nas doses de reforço. Utilizar serviço privado para imunização e o número de filhos foram associados à cobertura vacinal incompleta.

## Contribuições do estudo

**Principais resultados:**

A cobertura vacinal completa foi de pouco mais da metade da população, com diminuição para as vacinas que necessitam de dose de reforço. Utilizar serviço privado para imunização e o número de filhos foram associadas negativamente à cobertura vacinal completa.

**Implicações para os serviços:**

Fortalecermos a busca ativa de crianças com atraso vacinal nos territórios de saúde, principalmente nas famílias com um número maior de filhos, aquelas assistidas pela iniciativa privada.

**Perspectivas:**

Os serviços de saúde necessitam de estratégias inovadoras para aumentar a cobertura da vacinação infantil, sobretudo para o enfrentamento da insegurança e medo à vacinação decorrente de má informação.

## INTRODUÇÃO

Uma das principais medidas de prevenção de doenças é a imunização.^1^ A consolidação do Programa Nacional de Imunizações (PNI) no Brasil se deu pela eliminação da rubéola e da sua síndrome congênita, do tétano neonatal, da erradicação da poliomielite e da redução da transmissão da difteria, do tétano e da coqueluche.^1,2^ O PNI conquistou avanços no controle, redução e eliminação de doenças, mudando o cenário epidemiológico das doenças imunopreveníveis no país.^1,2^

Atualmente o PNI oferta mais de 20 vacinas de rotina atingindo todos os ciclos de vida. Para crianças até 2 anos de vida são ofertados 15 imunizantes, prevenindo mais de 19 doenças.^3^ Nos últimos anos, o Brasil tem registrado quedas importantes na cobertura vacinal da população, não atingindo as metas preconizadas pelo PNI.^4^ Com todos os esforços para aumentar a cobertura vacinal em nível nacional, ao comparar as coberturas de 2022 com 2023, a cobertura vacinal contra hepatite A passou de 73% para 79,5%. O primeiro reforço da vacina contra o pneumococo passou de 71,5% para 78% em 2023. A vacina contra poliomielite alcançou 74,6% de cobertura ante os 67,1% do ano 2022. Contudo, se mantém abaixo da meta preconizada pelo PNI.^5^

No município de Vitória, a tríplice viral no ano de 2022 passou de 92,33% para 88,27% em 2023, a meningocócica C para menores de 1 ano atingiu 87,25% em 2022 e 85,24% em 2023 e a vacina contra a poliomielite manteve-se em 91% nos respectivos anos. Além disso, a cobertura do primeiro reforço da vacina pneumocócica passou de 80,94% em 2022 para 78,59% em 2023.^6^

Diversos fatores podem interferir na cobertura vacinal. A disseminação de informações falsas sobre imunizantes, desinformação e o desconhecimento da gravidade das doenças imunopreveníveis podem desencadear baixa adesão à vacinação reduzindo as coberturas vacinais.^1,2,4,7,8^ Além disso, fatores relacionados às características da mãe, da criança e do contexto familiar podem influenciar nas coberturas vacinais, bem como fatores relacionados à estrutura dos serviços e à falta de insumos.^4,7-10^ A relevância do tema e a necessidade científica de estudos epidemiológicos, com abordagens metodológicas mais robustas, justificam a realização do estudo. Dessa forma, o objetivo do estudo foi estimar a prevalência do esquema vacinal completo de 12 a 24 meses de vida e analisar os fatores associados ao esquema vacinal completo. 

## MÉTODOS

### Desenho de estudo e cenário 

Trata-se de um inquérito de base populacional com amostragem por conglomerados realizado no município de Vitória, capital do estado do Espírito Santo, entre 16 de dezembro de 2020 e 4 de janeiro de 2021. O estudo integra o Inquérito Nacional de Cobertura Vacinal 2020.^11^

O município de Vitória está localizado na região metropolitana, possui a estimativa de 365.855 de habitantes, sendo 5,88% (21.406) de crianças entre 0 e 4 anos. Em dezembro de 2020, o município contava com 78 equipes vinculadas à Estratégia de Saúde da Família (ESF) com uma cobertura da ESF de 74,32% e da Atenção Básica de 93,8%. 

Existiam na cidade 43 salas de vacina: 28 localizadas em unidades públicas de saúde, um Centro de Referência de Imunobiológicos Especiais, 12 serviços privados de vacinação e 2 maternidades que aplicam a primeira dose de BCG e hepatite B em recém-nascidos.

### Participantes

A população do estudo foi composta pelas coortes de nascidos vivos de 2017 e 2018, registrados no Sistema de Informações sobre Nascidos Vivos (SINASC), sendo no total 9.252 nascidos vivos. Adotaram-se como critérios de inclusão crianças nascidas no município de Vitória durante os anos de 2017 e 2018 e com caderneta de vacinação. Os critérios de exclusão foram crianças que mudaram de município, óbitos e crianças com mães menores de 19 anos. 

### Tamanho amostral

O cálculo do tamanho amostral para o inquérito original seguiu como parâmetros uma prevalência estimada de cobertura vacinal de 70%, erro de estimativa igual a 5%, intervalo de confiança de 95% e efeito do desenho pelo uso de conglomerados igual a 1,4.

A amostragem se deu por estratos socioeconômicos, nos quais foram utilizados os setores censitários urbanos da cidade, segundo o censo demográfico de 2010. Após a identificação dos estratos socioeconômicos, foi realizada a estimativa de crianças residentes em cada setor censitário, por meio de georreferenciamento dos endereços contidos no SINASC e da projeção baseada na distribuição observada no Censo de 2010. Os setores censitários foram sorteados de modo sistemático para percorrer toda a área geográfica. Os entrevistadores percorreram a área buscando as crianças residentes até atingir o número preestabelecido para cada estrato.^11^

O processo de amostragem e detalhes do cálculo amostral do inquérito original está disponível na publicação do protocolo metodológico do Inquérito Nacional de Cobertura Vacinal 2020.^11^ Contudo, para este estudo foi realizado o cálculo do poder da amostra recrutada a partir da prevalência da cobertura vacinal encontrada com 24 meses de vida, considerando um erro alfa de 5%. O cálculo foi realizado utilizando-se o Stata v. 14.0. 

### Coleta de dados 

A coleta de dados foi através de entrevista dos pais ou responsáveis pela criança por questionário estruturado e a realização de uma imagem fotográfica do cartão de vacina da criança através de um dispositivo eletrônico. Os entrevistadores foram treinados e validados para a padronização da aplicação do questionário e realização da imagem fotográfica. A digitação das datas do cartão de vacina foi realizada posteriormente por dois profissionais com experiência em imunização, treinados e validados, a partir da imagem fotográfica do cartão de vacina. 

O questionário era composto pelos seguintes blocos: dados sociodemográficos da criança, reprodutivos da mãe, do domicílio e consumo da família, e sobre a vacinação da criança. Para os dados de imunização foi considerado o calendário vacinal do Ministério da Saúde de 2015, sendo:

- Ao nascer: bacilo de Calmette-Guérin (BCG) e hepatite B (HB);
- 2 meses: 1ª dose de difteria, tétano, pertússis, hepatite B, *Haemophilus influenzae* b (Penta), 1ª dose da vacina injetável contra poliomielite (VIP), 1ª dose da vacina pneumocócica conjugada 10 valente (Pneumo-10) e 1ª dose da vacina oral rotavírus humano (Rotavírus);
- 3 meses: 1ª dose da vacina meningocócica C conjugada (Meningo-C);
- 4 meses: 2ª dose Penta, 2ª dose VIP, 2ª dose Pneumo-10 e 2ª dose Rotavírus;
- 5 meses: 2º Meningo-C;
- 6 meses: 3ª dose Penta, 3ª dose VIP/vacina oral contra poliomielite (VOP);
- 12 meses: 1º reforço Pneumo-10, 1ª dose da vacina contra sarampo, caxumba e rubéola (SCR) e hepatite A (HA);
- 15 meses: 1ª dose da vacina contra difteria, tétano e pertússis, (DTP), 1º reforço VOP, 1º reforço Meningo-C, 2ª dose SCR e dose única varicela.

### Variáveis

A variável dependente do estudo foi a o esquema vacinal completo (sim; não). As crianças que receberam as doses recomendadas pelo calendário vacinal do Ministério da Saúde, independentemente da data de aplicação, foram consideradas como cobertura vacinal completa.

As variáveis independentes foram agrupadas em:

Características da família:
Beneficiários do Bolsa Família (sim; não);
Utilização do serviço privado de imunização (sim; não);
Aglomeração familiar (sim – ≥ 4 pessoas por cômodo; não – ≤ 3 pessoas por cômodo);
Mora com a avó (sim; não);
Renda familiar (em reais – até R$ 1.000; R$ 1.001 a R$ 3.000; R$ 3.001 a R$ 8.000; ≥ R$ 8.001).

Características da mãe:
Idade (19 a 29 anos; 30 a 39 anos; ≥ 40 anos);
Escolaridade (em anos de estudo – ≤ 8 anos; 9 a 12 anos; 13 a 15 anos; ≥ 16 anos);
Raça/cor da pele (branca; preta; parda; amarela/indígena);
Trabalho remunerado (sim; não);
Número de filhos (1 filho; 2 filhos; 3 filhos; ≥ 4 filhos);
Vive junto com o companheiro (sim; não);
Tipo de parto (vaginal; cesárea).

Características da criança:
Sexo (masculino; feminino);
Raça/cor da pele (branca; preta; parda; amarela/indígena);
Frequenta creche ou berçário (sim; não);
Ordem de nascimento (1º a nascer; 2º a nascer; 3º a nascer; ≥ 4º a nascer);

### Análise estatística 

Em virtude de a amostra ser estratificada e conglomerada por setor censitário, foram calculados os pesos amostrais para cada domicílio entrevistado, de modo a permitir a estimação sem viés dos parâmetros de interesse na população, além da utilização de procedimentos de alocação desproporcional (PPT). As etapas de cálculo e calibração dos pesos estão detalhadas no artigo operacional do Inquérito Nacional.^11^ As análises estatísticas foram realizadas com uso do Stata v. 14.0. O delineamento amostral foi considerado complexo, utilizando-se o conjunto de comandos svyset do Stata, que reduz a subestimação da variância das estimativas pontuais.^12^

 Foi estimada a prevalência do esquema vacinal completo até os 24 meses de vida e por cada imunizante com intervalo de confiança de 95% (IC_95_%). Também foram calculadas as frequências relativas e absolutas das variáveis independentes pela variável cobertura vacinal completa. 

Para verificar a associação das variáveis independentes com a prevalência cobertura vacinal completa, foi utilizada a regressão de Poisson de variância robusta. Inicialmente foi realizada uma análise bivariada (bruta). Em seguida foi realizada uma análise múltipla hierárquica na qual todas as variáveis de cada nível foram inseridas no modelo de uma só vez e mantidas nos níveis seguintes como ajustes se valor de p-valor ≤ 0,05. 

Para a análise múltipla, entendeu-se como níveis hierárquicos os seguintes grupos de variáveis, respectivamente: características da família, características da mãe e características da criança. Os resultados foram expressos pela medida de associação razão de prevalência (RP) e IC_95_%.

### Aspectos éticos 

O estudo foi aprovado pelos Comitês de Ética em Pesquisa com Seres Humanos do Instituto de Saúde Coletiva da Universidade Federal da Bahia, sob parecer número 3.366.818, em 4 de junho de 2019, com Certificado de Apresentação de Apreciação Ética (CAAE) 4306919.5.0000.5030; e da Irmandade da Santa Casa de São Paulo, sob parecer número 4.380.019, em 4 de novembro de 2020, com CAAE 39412020.0.0000.5479.

Todos os participantes foram orientados quantos aos procedimentos da pesquisa e manifestaram a vontade livre e desimpedida para participação no estudo assinando o termo de livre esclarecimento.

## RESULTADOS

O cálculo amostral do inquérito vacinal para o município de Vitória resultou em 904 crianças. Após o processo de seleção da amostra e entrevistas participaram do inquérito 788 crianças, uma perda de 12,8%. A cobertura vacinal, considerando todos os imunobiológicos aos 12 meses de vida, foi de 66,2% (IC_95_% 57,83;73,67) e a cobertura vacinal total aos 24 meses de vida foi de 57% (IC_95_% 50,98;62,98). Com a prevalência encontrada aos 24 meses de vida, obteve-se um poder da amostra de 97%.

Na Tabela 1 observa-se que a cobertura vacinal foi inferior a 90% para todos os imunobiológicos avaliados, tendo as primeiras doses das vacinas pentavalente (IC_95_% 78,21;94,66), pneumocócica (IC_95_% 78,22;94,67) e poliomielite (IC_95_% 77,95;94,44) apresentado a maior prevalência com 88% das crianças imunizadas com a primeira dose, enquanto o primeiro reforço da pneumocócica (72,4%, IC_95_% 61,76;81,03) e da poliomielite (78,3%, IC_95_% 66,46;86,89) e a segunda dose da tríplice viral (71,3%, IC_95_% 61,41;79,61) apresentaram a menor prevalência da cobertura vacinal. 

**Tabela 1:** Prevalência em (%) e com intervalos de confiança de 95% (IC95%) do esquema vacinal completo aos 12 e 24 meses de vida das crianças nascidas vivas de 2017 a 2018 em Vitória, Espírito Santo

Vacinas	Doses	% (I_C95_ **%**)
BCG	Dose única	79,77 (68,13;87,91)
Hepatite B	Dose inicial	78,97 (67,20;87,31)
Pentavalente	1ª Dose	88,86 (78,21;94,66)
Poliomielite	1ª Dose	88,57 (77,95;94,44)
Pneumocócica	1ª Dose	88,87 (78,22;94,67)
Rotavírus	1ª Dose	85,92 (75,26;92,44)
Meningocócica C	1ª Dose	87,96 (77,31;94,00)
Pentavalente	2ª Dose	85,49 (74,95;92,07)
Poliomielite	2ª Dose	85,56 (75,01;92,12)
Pneumocócica	2ª Dose	76,94 (64,56;85,94)
Rotavírus	2ª Dose	80,68 (69,13;88,92)
Meningocócica C	2ª Dose	85,06 (74,36;91,79)
Pentavalente	3ª Dose	81,80 (70,39;89,48)
Poliomielite	3ª Dose	82,08 (70,68;89,69)
Pneumocócica	1º Reforço	72,43 (61,76;81,03)
Meningocócica C	1º Reforço	79,63 (67,92;87,83)
Tríplice viral	1ª Dose	87,06 (76,52;93,29)
Hepatite A	Dose única	84,63 (73,37;91,67)
Tríplice viral	2ª Dose	71,37 (61,41;79,61)
Poliomielite	1º Reforço	78,37 (66,46;86,89)
Difteria, tétano e pertússis	1º Reforço	77,88 (65,95;86,48)
Varicela	Dose inicial	84,89 (73,66;91,86)
Cobertura vacinal aos 12 meses de vida	–	66,20 (57,83;73,67)
Cobertura vacinal aos 24 meses de vida	–	57,09 (50,98;62,98)

Quanto às características da família das crianças, observa-se que a maioria (57,2%, IC_95_% 41,99;71,17) utiliza o serviço privado para vacinação, não residem em locais com aglomeração familiar (95,1%, IC_95_% 91,10;96,63), não são beneficiários do Programa Bolsa Família (83,6%, IC_95_% 75,69;89,31), não moram junto com a avó (83,7%, IC_95_% 73,94;90,91) e possuem renda familiar mensal igual ou superior a R$ 8.001 (40,6%, IC_95_% 17,54;58,77) (Tabela 2). Ao se analisar essas características, considerando a condição da cobertura vacinal categorizada em completa e incompleta, observa-se um comportamento semelhante com o descrito para o total de crianças estudadas, destacando-se como diferenças a não utilização do serviço privado de vacinação e a renda familiar mensal entre R$ 1.001 e R$ 3.000 para o grupo com cobertura vacinal completa (Tabela 2).

**Tabela 2:** Distribuição em (%) e com intervalos de confiança de 95% (IC95%) da prevalência do esquema vacinal completo aos 12 e 24 meses de vida pelas características da família de crianças nascidas vivas de 2017 a 2018 em Vitória, Espírito Santo

Variável	Cobertura vacinal					
	**Incompleta**		**Completa**		**Total**	
	**n**	**% (IC**_95_ **%** **)**	**n**	**% (IC**_95_ **%** **)**	**n**	**% (IC**_95_ **%** **)**
**Serviço privado para vacinação**						
Não	121	13,12 (8,61;19,49)	230	29,63 (20,07;41,38)	351	42,75 (28,78;57,92)
Sim	175	29,68 (21,57;39,31)	258	27,57 (19,92;36,80)	433	57,25 (41,99;71,17)
**Aglomeração familiar**						
Não	281	41,28 (35,00;47,65)	463	54,00 (48,40;59,51)	744	95,18 (91,10;96,63)
Sim	17	1,73 (0,91;3,26)	23	3,09 (1,69;5,58)	40	4,82 (3,37;8,90)
**Beneficiário do Bolsa Família**						
Não	252	38,51 (32,00;45,47)	393	45,14 (39,55;50,85)	645	83,65 (75,69;89,31)
Sim	47	4,42 (2,75;7,03)	95	11,93 (7,55;18,35)	142	16,35 (10,66;24,25)
**Mora junto com a avó**						
Não	256	35,58 (28,76;43,50)	404	47,92 (43,00;52,88)	660	83,71 (73,94;90,91)
Sim	42	7,07 (3,07;15,45)	85	9,21 (5,72;14,52)	127	16,29 (9,72;25,97)
**Renda familiar mensal (R$)**						
≤ R$ 1.000	42	5,36 (3,08;9,17)	79	12,36 (7,01;20,87)	121	17,72 (9,48;24,21)
R$ 1.001 a R$ 3.000	44	5,18 (2,95;8,97)	108	15,58 (9,88;23,72)	154	20,77 (12,21;26,06)
R$ 3.001 a R$ 8.000	74	10,65 (6,35;17,32)	108	10,18 (6,19;16,30)	182	20,83 (12,13;26,36)
≥ R$ 8.001	98	17,43 (8,10;33,57)	161	23,25 (14,39;35,32)	259	40,68 (17,54;58,77)

No que se refere às características das mães, nota-se uma maior prevalência de mães com idade entre 30 e 39 anos (56,7%, IC_95_% 40,97;71,28), 16 ou mais anos de estudo (63%, IC_95_% 48,47;74,41), da raça/cor da pele branca (50,8%, IC_95_% 44,96;56,64), com trabalho remunerado (67,3%, IC_95_% 54,22;78,20), somente um filho (47,1%, IC_95_% 41,42;53,03), vivendo com o companheiro (83,4%, IC_95_% 75,52;89,23) e que tiveram parto cesárea (71%, IC_95_% 59,09;80,64). Comparando os estratos de cobertura vacinal completa e incompleta, notam-se características similares entre os dois grupos (Tabela 3).

**Tabela 3:** Distribuição da prevalência em (%) e com intervalos de confiança de 95% (IC95%), do esquema vacinal completo aos 12 e 24 meses de vida pelas características das mães de crianças nascidas vivas em 2017 e 2018 em Vitória, Espírito Santo

Variável	Cobertura vacinal					
	**Incompleta**		**Completa**		**Total**	
	**n**	**% (IC**_95_ **%** **)**	**n**	**% (IC**_95_ **%** **)**	**n**	**% (IC**_95_ **%** **)**
**Idade**						
19 a 29	61	7,39 (4,68;11,48)	108	15,75 (10,00;23,92)	169	23,14 (15,30;33,41)
30 a 39	167	26,52 (18,35;36,67)	255	30,25 (22,76;38,96)	422	56,76 (40,97;71,28)
≥ 40	71	9,03 (4,52;17,24)	125	11,07 (7,23;16,59)	196	20,10 (12,60;30,52)
**Escolaridade (anos)**						
≤ 8	12	1,08 (0,51;2,26)	20	1,82 (0,98;3,34)	32	2,89 (1,64;5,04)
9 a 12	33	4,04 (2,32;6,95)	47	6,71 (3,93;11,21)	80	10,75 (6,67;16,85)
13 a 15	57	5,60 (3,44;8,99)	127	17,76 (11,29;26,81)	184	23,36 (15,34;33,83)
≥ 16	195	32,49 (24,76;41,30)	287	30,51 (23,12;39,07)	482	63,00 (48,47;74,41)
**Raça/cor da pele**						
Branca	168	21,49 (13,04;33,32)	239	29,32 (21,82;38,13)	407	50,81 (44,96;56,64)
Preta	37	4,24 (2,44;7,28)	64	7,36 (4,51;11,80)	101	11,61 (7,45;17,65)
Parda	92	17,47 (8,78;31,77)	170	19,38 (12,89;28,08)	262	36,85 (30,66;43,50)
Amarela/indígena	1	0,003 (0,001;0,23)	8	0,07 (0,02;1,68)	9	0,73 (0,31;1,71)
**Trabalho remunerado**						
Não	103	11,77 (7,64;17,71)	194	20,88 (13,89;30,17)	297	32,66 (21,79;45,76)
Sim	195	31,46 (23,78;40,31)	287	35,88 (28,67;43,79)	482	67,34 (54,22;78,20)
**Número de filhos**						
1	127	17,34 (10,18;27,96)	208	29,85 (22,21;38,80)	335	47,19 (41,42;53,03)
2	119	20,09 (11,67;32,36)	198	19,21 (12,87;27,66)	317	39,29 (33,09;45,86)
3	33	3,51 (2,01;6,08)	54	5,37 (3,31;8,58)	87	8,87 (5,66;13,64)
≥ 4	20	2,00 (0,94;4,21)	28	2,65 (1,44;4,84)	48	4,65 (2,75;7,75)
**Vive com companheiro**						
Não	50	5,68 (3,39;9,36)	86	10,84 (7,01;16,38)	136	16,51 (10,77;24,48)
Sim	247	37,52 (30,07;44,87)	397	45,96 (40,66;51,36)	644	83,49 (75,52;89,23)
**Tipo de parto**						
Cesárea	194	32,51 (24,81;41,28)	323	38,53 (32,65;44,76)	517	71,04 (59,09;80,64)
Vaginal	101	10,24 (6,54;15,68)	166	18,72 (12,52;27,04)	267	28,96 (19,36;40,91)

Avaliando o total das crianças estudadas, observa-se que a maior parte era do sexo masculino (53,3%, IC_95_% 48,01;58,54), da raça/cor da pele branca (55,8%, IC_95_% 49,21;62,19), frequentava creche (76%, IC_95_% 64,72;84,58) e foram as primeiras na ordem de nascimento (55,1%, IC_95_% 49,57;60,63). Não foram observadas diferenças nas características das crianças entre os grupos de cobertura vacinal completa e incompleta, ou em relação ao total das crianças avaliadas, como representado na Tabela 4.

**Tabela 4:** Distribuição da prevalência em (%) e com intervalos de confiança de 95% (IC95%), do esquema vacinal completo aos 12 e 24 meses de vida pelas características das crianças nascidas vivas em 2017 e 2018 em Vitória, Espírito Santo

Variável	Cobertura vacinal					
	**Incompleta**		**Completa**		**Total**	
	**n**	**% (IC**_95_ **%** **)**	**n**	**% (IC**_95_ **%** **)**	**n**	**% (IC**_95_ **%** **)**
**Sexo**						
Feminino	137	16,71 (9,99;26,62)	228	29,98 (22,58;38,59)	365	46,69 (41,46;51,99)
Masculino	162	26,20 (17,82;36,77)	261	27,11 (18,41;37,99)	423	53,31 (48,01;58,54)
**Raça/cor da pele**						
Branca	185	22,22 (13,68;34,01)	283	33,58 (26,79;41,12)	468	55,80 (49,21;6219)
Preta	16	2,14 (1,04;4,32)	46	4,97 (2,93;8,31)	62	7,11 (4,38;11,33)
Parda	97	18,47 (9,79;32,11)	156	18,36 (12,04;26,99)	253	36,83 (29,98;44,26)
Amarela/indígena	1	0,01 (0,01;0,73)	3	0,16 (0,01;0,55)	4	0,26 (0,04;0,78)
**Frequenta creche**						
Não	63	7,59 (4,39;12,81)	83	16,38 (7,77;31,30)	146	23,97 (15,42;35,28)
Sim	236	35,32 (28,14;43,23)	406	40,71 (27,40;55,53)	642	76,03 (64,72;84,58)
**Ordem de nascimento**						
1º	167	20,32 (12,38;31,53)	263	34,84 (28,01;42,36)	430	55,16 (49,57;60,63)
2º	89	17,65 (9,09;31,47)	162	16,18 (10,77;23,59)	251	33,82 (26,99;41,40)
3º	24	2,86 (1,53;5,28)	39	3,92 (2,36;6,43)	63	6,77 (4,25;10,64)
≥ 4º	18	1,85 (0,86;3,93)	24	2,39 (1,26;4,51)	42	4,24 (2,48;7,16)

A análise bruta das características da família, da mãe e da criança mostrou uma associação significativa com a cobertura vacinal completa nos seguintes fatores: utilização do serviço privado para realização da imunização (RP 0,53; IC_95_% 0,40;0,69), ser beneficiário do Programa Bolsa Família (RP 1,28; IC_95_% 1,11;1,46), possuir renda familiar mensal ≥ R$ 8.001,00 (RP 0,66; IC_95_% 0,53;0,82), idade da mãe de 30 a 49 anos (RP 0,68; IC_95_% 0,55;0,84) e ≥ 40 anos (RP 0,65; IC_95_% 0,46;0,92), ter trabalho remunerado (RP 0,76; IC_95_% 0,62;0,94), e tipo de parto por via vaginal tido parto normal (RP 1,23; IC_95_% 1,04;1,47). Na análise múltipla hierárquica, a cobertura vacinal completa foi associada à utilização do serviço privado de imunização (RP 0,66; IC_95_% 0,51;0,86), mãe possui três filhos (RP 0,67; IC_95_% 0,45;0,98) ou ≥ 4 filhos (RP 0,55; IC_95_% 0,32;0,94) (Tabela 5).

**Tabela 5:** Fatores associados à prevalência do esquema vacinal completo aos 12 e 24 meses de vida na regressão Poisson bruta e multinível com intervalos de confiança de 95%, das crianças nascidas vivas em 2017 e 2018 em Vitória, Espírito Santo

Variável	RP Modelo bruto (IC95%)	p-valor	RP Modelo multinível (IC95%)	p-valor
Características da família				
**Serviço privado para vacinação**		< 0,001		0,002
Não	1,00		1,00	
Sim	0,53 (0,40;0,69)		0,66 (0,51;0,86)	
**Aglomeração familiar**		0,392		0,644
Não	1,00		1,00	
Sim	1,14 (0,84;1,54)		0,94 (0,73;1,20)	
**Beneficiário do Bolsa Família**		< 0,001		0,720
Não	1,00		1,00	
Sim	1,28 (1,11;1,46)		1,02 (0,89;1,16)	
**Mora junto com a avó**		0,663		0,496
Não	1,00		1,00	
Sim	1,05 (0,82;1,33)		1,05 (0,90;1,23)	
**Renda familiar mensal (R$)**		< 0,001		0,065
≤ R$ 1.000	1,00		1,00	
R$ 1.001 a R$ 3.000	1,08 (0,87;1,35)		1,12 (0,89;1,40)	
R$ 3.001 a R$ 8.000	0,63 (0,45;1,35)		0,75 (0,54;1,04)	
≥ R$ 8.001	0,66 (0,53;0,82)		0,95 (0,68;1,32)	
**Características da mãe**				
**Idade (anos)**		< 0,001		0,153
19 a 29	1,00		1,00	
30 a 49	0,68 (0,55;0,84)		1,24 (0,95;1,62)	
≥ 40	0,65 (0,46;0,92)		1,25 (0,89;1,75)	
**Escolaridade (anos)**		< 0,001		0,078
≤ 8	1,00		1,00	
9 a 12	1,03 (0,73;1,44)		0,98 (0,67;1,44)	
13 a 15	1,21 (0,90;1,62)		1,07 (0,74;1,55)	
≥ 16	0,62 (0,43;0,88)		0,65 (0,40;1,05)	
**Raça/cor da pele**		0,082		0,637
Branca	1,00		1,00	
Preta	1,22 (0,79;1,87)		0,82 (0,57;1,18)	
Parda	0,93 (0,41;2,12)		0,79 (0,46;1,36)	
Amarela/indígena	1,48 (0,86;2,56)		0,97 (0,52;1,83)	
**Trabalho remunerado**		0,012		0,303
Não	1,00		1,00	
Sim	0,76 (0,62;0,94)		0,90 (0,73;1,09)	
**Número de filhos**		0,491		0,045
1	1,00		1,00	
2	0,72 (0,35;1,49)		0,70 (0,39;1,25)	
3	1,02 (0,69;1,51)		0,67 (0,45;0,98)	
≥ 4	0,96 (0,58;1,58)		0,55 (0,32;0,94)	
**Vive com companheiro**		0,003		0,096
Não	1,00		1,00	
Sim	0,76 (0,64;0,91)		1,13 (0,97;1,30)	
**Tipo de parto**		0,014		0,928
Cesárea	1,00		1,00	
Vaginal	1,23 (1,04;1,47)		1,01 (0,82;1,30)	
**Características da criança**				
**Sexo**		0,390		0,280
Feminino	1,00		1,00	
Masculino	0,76 (0,41;1,41)		0,83 (0,59;1,16)	
**Raça/cor da pele**		0,257		0,584
Branca	1,00		1,00	
Preta	1,26 (0,84;1,87)		0,82 (0,58;1,15)	
Parda	0,88 (0,41;1,88)		0,81 (0,58;1,14)	
Amarela/indígena	1,17 (0,46;2,99)		1,13 (0,41;3,13)	
**Frequenta creche**		0,340		0,339
Não	1,00		1,00	
Sim	0,73 (0,11;2,52)		0,69 (0,42;1,13)	
**Ordem de nascimento**		0,786		0,218
1º	1,00		1,00	
2º	0,75 (0,35;1,62)		0,82 (0,55;1,22)	
3º	1,00 (0,67;1,49)		0,68 (0,45;1,03)	
≥ 4º	0,99 (0,60;1,61)		0,71 (0,46;1,10)	

## DISCUSSÃO

Nosso estudo apontou uma baixa cobertura vacinal entre os nascidos vivos de 2017 e 2018 no município de Vitória. Quando a análise foi entre os maiores de 1 ano de vida, a cobertura vacinal apresentou uma queda relevante. Observou-se, também, que as primeiras doses das vacinas pentavalente, pneumocócica e poliomielite apresentam as maiores coberturas na população, contudo, o primeiro reforço da pneumocócica e da poliomielite e a segunda dose da tríplice viral apresentaram as menores coberturas vacinais. No que tange aos fatores associados à cobertura vacinal completa, a utilização do serviço privado de imunização e a maior quantidade filhos mostraram-se negativamente associadas com a cobertura completa da vacinação.

Embora os resultados deste estudo tenham sua importância para a saúde pública, devem-se destacar as limitações. A pandemia de covid-19 dificultou o acesso aos domicílios das famílias selecionadas pelo inquérito. Bem como aumentou o receio de receber a equipe do estudo. Para isso, utilizou-se das mídias sociais e entrevistas aos principais meios de comunicação local. Esta dificuldade no acesso aos domicílios pode ter provocado uma porcentagem maior de um estrato social da população. Todavia, acreditamos que após o ajuste no modelo não foi observada interferência no resultado. Além disso, o delineamento do estudo, o processo de amostragem das crianças e o tamanho amostral permitiu um poder ao estudo para detectar diferenças entre os grupos estudados. 

Os resultados do inquérito descreveram uma cobertura vacinal bem abaixo do estabelecido pela Organização Mundial da Saúde e pactuado pelo PNI brasileiro, que é de 95% de cobertura.^3,4,13^ Nesse aspecto, a baixa cobertura vacinal pode reintroduzir e recrudescer doenças já controladas e eliminadas, como o sarampo, que desde 2018 o Brasil vem enfrentando a sua reintrodução no território nacional.^14-16^

Um estudo ecológico, realizado com dados secundários, descreveu uma correlação inversamente proporcional entre o aumento do número de casos de sarampo e a diminuição da cobertura vacinal no Brasil.^17^ No ano de 2018, no Brasil, a cobertura vacinal contra sarampo era de 67%, ano em que ocorreram 10.326 casos da doença.^17^ Sendo assim, a baixa cobertura da vacina contra sarampo, encontrada no estudo, demonstra um cenário emergente para a ocorrência de casos autóctones da doença. 

 Com os resultados deste inquérito, observa-se uma queda considerável na terceira dose da poliomielite em relação à primeira dose, o que sugere a compreensão de que a cobertura tende a ser menor em vacinas que necessitam de reforço em comparação às de dose única.^18^ No inquérito vacinal nacional, todos os municípios estudados apresentaram uma queda nas coberturas das vacinas que necessitam de mais de uma dose, quando compare-se a cobertura da primeira dose com as demais.^11^

Os resultados apresentados sinalizam menor chance de cobertura vacinal completa em crianças que acessam o serviço privado para a vacinação. Pode-se considerar como possível explicação a ocorrência de atividades estratégicas e as iniciativas que são apresentadas para manter a sala de vacina aberta durante o horário de funcionamento da unidade; evitar barreiras de acesso como a não obrigatoriedade do comprovante de residência para vacinação, bastando apenas o cartão do SUS; aproveitar as oportunidades de vacinação como consultas ou outros procedimentos na unidade de saúde para verificar a situação vacinal, além das campanhas de vacinação que contribuam para a realização de cobertura vacinal amplamente preconizadas pelo PNI nas unidades básicas de saúde e, claro, a contribuição da ESF na busca ativa das crianças com carteira de vacinação atrasada.^19-21^

Pode-se destacar também a continuidade da oferta universal de vacinas na rede pública e seu acesso facilitado à população, dada a extensa rede nacional de serviços básicos de saúde, juntamente com outras ações de vigilância, que são fundamentais e imprescindíveis para alcançar o objetivo de eliminação e controle de doenças como varíola, difteria, poliomielite e sarampo.^4^

As crianças de família com renda mais elevada tiveram a menor prevalência de terem a carteira de vacinação completa na análise bruta, contrapondo as famílias que recebiam algum auxílio governamental. As crianças de família com menor poder aquisitivo residem em bairros mais periféricos e com boa cobertura da ESF no município de Vitória. Portanto, acreditamos que a ESF e o auxílio governamental compreendem estratégias de saúde e políticas públicas importantes para a vacinação.^22,23^ Em um cenário mundial de *fake news* sobre as vacinas, a ESF, sobretudo os agentes comunitários de saúde da família na realização das visitas domiciliares, exerce um papel fundamental no enfrentamento à desinformação e busca ativa das crianças em atraso vacinal.^24,25^

Quanto ao recebimento do auxílio governamental, deve-se, ainda, ponderar o evidente risco de incremento na diminuição da cobertura vacinal em razão das modificações das condicionantes associadas ao benefício social promovidas recentemente no Brasil, desvinculando-se o recebimento do benefício, batizado de Auxílio Brasil, de diversas contrapartidas condicionantes por parte dos responsáveis familiares, entre eles a vacinação em dia conforme o PNI, das crianças que compõem o núcleo familiar, levando ao desestímulo à vacinação e risco de diminuição da cobertura vacinal.

No nosso estudo, ter três ou mais filhos mostrou-se negativamente associado com a cobertura completa da vacinação. Acreditamos que a menor prevalência de ter a vacinação completa pode ser devido ao menor tempo disponível da mãe/responsável para o cuidado integral da criança e, claro, com mais gastos na família, declina os cuidados e a priorização da saúde.^26^

Embora a pesquisa investigue preponderantemente a relação entre a genitora e a cobertura vacinal das crianças, não é silente a uma perspectiva de gênero relevante a respeito desta escolha metodológica. A responsabilização pelos cuidados com as crianças é dever de todos os responsáveis, conforme preconizado pela Constituição Federal e pelas normativas específicas, cabendo à mãe, pai ou qualquer outro responsável prover os cuidados com a saúde, como a adesão das campanhas de vacinação.^27,28^ Todavia, da mesma forma que é importante reconhecer que no campo normativo há uma obrigação igualitária da responsabilização pelos cuidados – e, logo, pela vacinação – das crianças entre pais e mães, também é importante reconhecer que ainda vivemos em uma sociedade estruturalmente machista, que provoca uma responsabilização sobreposta de funções às mulheres, especialmente quando mães, recaindo culturalmente sobre elas o dever social e comunitário de cumprir com os cuidados parentais.^29,30^

Este estudo possibilitou identificar a baixa cobertura vacinal, a diminuição da cobertura das vacinas que necessitam de dose de reforço e aponta, epidemiologicamente, os fatores associados com a cobertura da vacinação completa. Por conseguinte, os achados deste estudo permitem-nos reforçar a necessidade de estratégias inovadoras para aumentar a cobertura da vacinação infantil, sobretudo para o enfrentamento da insegurança e medo à vacinação decorrente de má informação. Nesse ínterim, os agentes comunitários de saúde devem ser capacitados para a busca ativa de crianças com atraso vacinal nos territórios de saúde, principalmente nas famílias com um número maior de filhos, aquelas assistidas pela iniciativa privada, com menor renda, bem como disseminar informações positivas sobre a vacinação, além de ajudar a combater as informações inverídicas sobre vacinação no território.
